# Severe Respiratory Illness Outbreak Associated with Human Coronavirus NL63 in a Long-Term Care Facility

**DOI:** 10.3201/eid2410.180862

**Published:** 2018-10

**Authors:** Julie Hand, Erica Billig Rose, Andrea Salinas, Xiaoyan Lu, Senthilkumar K. Sakthivel, Eileen Schneider, John T. Watson

**Affiliations:** Louisiana Department of Health, Baton Rouge, Louisiana, USA (J. Hand, A. Salinas);; Centers for Disease Control and Prevention, Atlanta, Georgia, USA (E.B. Rose, X. Lu, S.K. Sakthivel, E. Schneider, J.T. Watson)

**Keywords:** outbreak, coronavirus, long-term care facility, respiratory infections, Louisiana, USA, United States, NL63, viruses, lower respiratory tract infection

## Abstract

We describe an outbreak of severe respiratory illness associated with human coronavirus NL63 in a long-term care facility in Louisiana in November 2017. Six of 20 case-patients were hospitalized with pneumonia, and 3 of 20 died. Clinicians should consider human coronavirus NL63 for patients in similar settings with respiratory disease.

Human coronaviruses (HCoVs) OC43, 229E, NL63, and HKU1 are frequently associated with upper respiratory tract infection but can also cause lower respiratory tract infections (LRTIs), such as pneumonia or bronchitis. Transmission of these viruses primarily occurs through respiratory droplets and indirect contact with secretions from infected persons. Signs and symptoms of illness often include runny nose, headache, cough, sore throat, and fever. LRTI occurs less frequently, but young children, older adults, and persons who are immunosuppressed appear to be at higher risk for these types of infections (*1*–*3*).

A wide range of respiratory viruses are known to circulate in long-term care facilities (LTCFs) and contribute to respiratory illness in the residents who live in them (*4*). Although outbreaks of HCoV-OC43 have been described among elderly populations in long-term care settings (*5*), outbreaks of severe respiratory illness associated with HCoV-NL63 have not, to our knowledge, been documented in LTCF settings.

On November 15, 2017, the Louisiana Department of Health (Baton Rouge, Louisiana, USA) was notified of a possible outbreak of severe respiratory illness by a representative of an LTCF that provides nursing home care and short-term rehabilitation services to 130 residents. At the time of notification, the facility reported 11 residents with chest radiograph–confirmed pneumonia. For this investigation, we defined a case-patient as any LTCF resident with respiratory tract symptoms of new onset in November 2017, and we considered LRTI diagnoses that were based on clinical or radiologic evidence. During November 1–18, a total of 20 case-patients (60% male) of a median age of 82 (range 66–96) years were identified. The number of cases of respiratory illness peaked in mid-November. The most common symptoms were cough (95%) and chest congestion (65%). Shortness of breath, wheezing, fever, and altered mental status were also reported (Table). Sixteen (80%) case-patients had abnormal findings on chest radiograph; pneumonia was noted in 14. All case-patients had concurrent medical conditions; the most common were heart disease (70%, 14/20), dementia (65%, 13/20), hypertension (40%, 8/20), diabetes (35%, 7/20), and lung disease (35%, 7/20). Six (30%) case-patients required hospitalization; all had chest radiograph–confirmed pneumonia. Hospitalized LRTI case-patients demonstrated shortness of breath (50% vs. 10%), wheezing (50% vs. 0%), and altered mental status (33% vs. 0%) more frequently than did nonhospitalized LRTI case-patients (Table).

**Table:** Signs and symptoms reported by case-patients with severe respiratory illness during outbreak associated with human coronavirus NL63 in long-term care facility, by hospitalization status, Louisiana, USA, November 1–18, 2017

Sign or symptom	No. (%) case-patients with severe respiratory illness, N = 20	Case-patients with lower respiratory tract infection, no. (%)		
		Total, n = 16	Hospitalized, n = 6	Nonhospitalized, n = 10
Cough	19 (95)	15 (94)	5 (83)	10 (100)
Chest congestion	13 (65)	9 (56)	1 (17)	8 (80)
Shortness of breath	5 (25)	4 (25)	3 (50)	1 (10)
Wheezing	3 (15)	3 (19)	3 (50)	0
Fever*	3 (15)	3 (19)	1 (17)	2 (20)
Altered mental status	2 (10)	2 (13)	2 (33)	0

*Fever as recorded in patient medical charts.

We performed molecular diagnostic viral testing on nasopharyngeal specimens from 13 case-patients by real-time PCR at the Louisiana State Public Health Laboratory (Baton Rouge, Louisiana, USA) and the Centers for Disease Control and Prevention (Atlanta, Georgia, USA). Of the 13 available specimens, HCoV-NL63 was detected in 7 (54%); rhinovirus was co-detected in 2 specimens. Of the 6 specimens negative for HCoV-NL63, 1 was positive for rhinovirus, and 1 was positive for parainfluenza virus 1. For the 6 case-patients hospitalized with pneumonia, median length of hospital stay was 4 (range 1–5) days; none of these case-patients were mechanically ventilated or admitted to the intensive care unit. Nasopharyngeal specimens were available from 2 of the hospitalized case-patients, and HCoV-NL63 was detected in both. HCoV-NL63 respiratory infection was considered a contributory cause in the deaths of 3 case-patients (1 hospitalized and 2 nonhospitalized) that occurred 10–36 days after illness onset. The concurrent medical conditions of those who died included dementia, cardiovascular disease, cancer, multiple sclerosis, Parkinson disease, diabetes mellitus, hypertension, asthma, and chronic kidney disease.

Infection control measures, including adherence to standard and droplet precautions for symptomatic residents, reviewing hand and personal hygiene policies, and enhanced environmental cleaning, were implemented in the LTCF on November 15. All residents were monitored daily for the onset of respiratory symptoms. The case-patients resided in rooms throughout the facility. However, residents often shared rooms, walked throughout the facility, and spent much of their time in shared areas (e.g., gym, dining rooms, and recreational rooms). Because all case-patients had visited the gym at the facility for recreation or physical therapy before becoming ill, environmental cleaning of this area was performed. No new cases among residents were identified after November 18, 2017, and no facility staff members reported respiratory symptoms during this outbreak.

The use of molecular diagnostics and respiratory virus panels has become more common, enabling specific HCoVs to be more easily identified. In the United States, HCoV respiratory infections commonly occur in the fall and winter, and annual variations are noted in patterns of circulation of individual HCoVs (*6*). At the time of this outbreak, national surveillance data from the National Respiratory and Enteric Virus Surveillance System indicated that HCoV-NL63 was the predominant circulating HCoV type.

This outbreak demonstrates that HCoV-NL63 can be associated with severe respiratory illness in LTCF residents. Clinicians and public health practitioners should consider HCoV-NL63 in patients with similar clinical presentations in these settings.
